# Chemosensitivity of three patient-derived primary cultures of canine pericardial mesothelioma by single-agent and combination treatment

**DOI:** 10.3389/fvets.2023.1267359

**Published:** 2023-11-02

**Authors:** Rina Nabeta, Ami Kanaya, Mohamed Elbadawy, Tatsuya Usui, Tetsuya Furuya, Kazuhiko Suzuki, Tsuyoshi Uchide

**Affiliations:** ^1^Laboratory of Veterinary Molecular Pathology and Therapeutics, Faculty of Agriculture, Tokyo University of Agriculture and Technology, Fuchu, Tokyo, Japan; ^2^Laboratory of Veterinary Pharmacology, Faculty of Agriculture, Tokyo University of Agriculture and Technology, Fuchu, Tokyo, Japan; ^3^Department of Pharmacology, Faculty of Veterinary Medicine, Benha University, Benha, Egypt; ^4^Department of Pathology, College of Veterinary Medicine, University of Georgia, Athens, GA, United States; ^5^Laboratory of Veterinary Infectious Diseases, Faculty of Agriculture, Tokyo University of Agriculture and Technology, Fuchu, Tokyo, Japan; ^6^Laboratory of Veterinary Toxicology, Faculty of Agriculture, Tokyo University of Agriculture and Technology, Fuchu, Tokyo, Japan

**Keywords:** pericardial mesothelioma, canine, chemotherapy, carboplatin, gemcitabine, combination therapy, primary cultures, xenograft

## Abstract

**Introduction:**

Canine mesothelioma is a rare malignant tumor that mostly affects body cavities, such as the pericardial and pleural cavities. Chemotherapy plays a crucial role in the treatment of canine mesotheliomas. We aimed to compare the antitumor effects of single-agent and combination chemotherapeutic agents on patient-derived primary cultures of canine pericardial mesothelioma established in this study. We planned to generate xenograft models for future studies.

**Material and methods:**

Effusion samples were collected from three dogs with histologically diagnosed pericardial mesothelioma and used for primary culture. Cultured cells were characterized by immunostaining for pan-cytokeratin AE1/AE3, vimentin, Wilms' tumor suppressor gene 1 (WT1), and cytokeratin 5 (CK5). To assess the tumorigenic properties of cells in the effusion and generate a xenograft model, the cell suspension was injected into a severe combined immunodeficient (SCID) mouse either subcutaneously (SC) or intraperitoneally (IP). Lastly, chemosensitivity of established primary cultures against four drugs, doxorubicin, vinorelbine, carboplatin, and gemcitabine, by single-agent treatment as well as combination treatment of carboplatin at a fixed concentration, either 10 or 100 μM, and gemcitabine at different concentrations ranging from 0–1000 μM was assessed by cell viability assay.

**Results:**

Primary cultures were successfully generated and characterized by dual positivity for AE1/AE3 and vimentin and positive staining for WT-1 and CK5, confirming the mesothelial origin of the cells. In the xenograft models, SC mouse developed a subcutaneous mass, whereas IP mouse developed multiple intraperitoneal nodules. The masses were histopathologically consistent with mesotheliomas. The chemosensitivity assay revealed that carboplatin had the highest anti-tumor effects among the four tested single-agent treatments. Furthermore, carboplatin at 100 μM combined with gemcitabine at clinically relevant doses demonstrated the augmented anti-tumor effects compared to single-agent treatment.

**Discussion and conclusion:**

Primary cultures and xenograft models generated in this study could be useful tools for *in vitro* and *in vivo* studies of canine mesothelioma. Carboplatin is a highly effective chemotherapeutic agent against canine mesothelioma when used as a sole agent and in combination with gemcitabine.

## 1. Introduction

Canine mesothelioma is a rare malignant tumor arising from the mesothelial cells that line the surface of body cavities. Canine mesotheliomas mostly involve the pericardial and pleural cavities (1–3). Dogs with mesothelioma often exhibit non-specific clinical signs, such as lethargy, anorexia, weight loss, dyspnea, or heart failure due to fluid accumulation in the affected body cavities (4–6). Imaging diagnosis is highly effective in detecting effusion in body cavities; however, it often fails to determine the cause of effusion as mesothelioma because they seldom, if ever, develop a discrete large mass enough to be detected by this less invasive modality until the later stages of the disease (2, 4, 7–10). Owing to the lack of specific clinical signs and the difficulty of early diagnosis using routine diagnostic tools, complete surgical resection of the mass is not possible in most cases because of disease progression at the time of diagnosis (6, 11).

Chemotherapy plays a crucial role in the treatment of unresectable canine mesotheliomas, as in humans, because of the tendency of mesotheliomas to form diffuse small nodular lesions throughout the body cavities, and the difficulty in targeting the surface of the body cavities by radiotherapy, which is another useful therapeutic option for unresectable solid tumors. Nevertheless, *in vitro* and *in vivo* studies on the efficacy of chemotherapy and standard treatment protocols for canine mesothelioma are sparse. Indeed, most published cases of canine mesothelioma empirically used classic chemotherapeutic agents based on previous single-case reports or small case series, where longer survival was achieved compared with historical non-treated records (6, 11–15). Recently, a review article was published on the outcomes of dogs treated with chemotherapy based on 40 retrospective cases (11). In that study, the effectiveness of chemotherapy was confirmed, as it was the sole treatment independently associated with survival in the cohort. However, the retrospective nature of the study limits the ability to determine the most effective chemotherapeutic agent for mesothelioma.

The paucity of fundamental studies is partially due to the lack of basic research tools, such as cell lines and preclinical animal models of canine mesothelioma. Thus, the objectives of this study were to (i) establish and characterize primary cultures derived from canine patients with pericardial mesothelioma as an *in vitro* experimental model, (ii) generate xenograft animal models for future preclinical *in vivo* studies, and (iii) perform a chemosensitivity assay using patient-derived primary cultures to determine the efficacy of chemotherapy and compare the anti-neoplastic effects of selected chemotherapeutic agents currently available for veterinary patients against canine pericardial mesotheliomas. This study aimed to establish an experimental model and provide a scientific basis for using chemotherapeutic agents and potential treatment protocols.

## 2. Material and methods

### 2.1. Sample collection

Effusion samples were obtained in a sterile manner via ultrasound-guided pericardiocentesis or thoracentesis using a 23-gauge needle from dogs that were referred to the Animal Medical Center, Tokyo University of Agriculture and Technology, between 2018 and 2019 for the tentative diagnosis of pericardial mesothelioma. Samples included in this study were obtained from dogs with a confirmed diagnosis through histopathology, whereas those without histopathological confirmation were excluded from the study. Owners of all dogs included in this study provided both verbal and written informed consent. Animal ethics approval was obtained, and the entire study was carried out in accordance with the recommendations of the Guidelines by the Clinical Research Ethics Committee of the Tokyo University of Agriculture and Technology (no. 0016017).

### 2.2. Cell culture

The collected effusion samples were immediately processed for primary cell culture. The cells in the effusion were isolated by centrifugation at 600 g for 3 min and washed three times with phosphate buffered saline (PBS). When a sample had prominent blood contamination it was incubated with RBC lysis buffer (Red Blood Cell Lysing Buffer Hybri-Max, Sigma Life Science) for 15 min at 37 °C to remove red blood cells. After the final centrifugation, cell pellets containing neoplastic cells were resuspended in culture medium, RPIM-1604 supplemented with 10% inactivated fetus bovine serum (FBS) and 100 μg/ml Primocin (InvivoGen), at the concentration of 1 × 10^6^ cells/ml. A culture medium with the same composition was used for all experiments in this study. A total of 10 mL of the cell suspension was seeded in a 75 cm^2^ culture flask. Cells were grown in a humidified incubator at 37 °C and 5% CO_2_. The cells were passaged at 70–80% confluency.

### 2.3. Immunostaining

Immunostaining was performed to characterize primary cultures of canine pericardial mesothelioma. First, the cultured cells were washed in PBS, detached from the flask by treatment with a 0.25% trypsin/ethylenediaminetetraacetic acid (EDTA) solution for 10 min at room temperature, and suspended in the culture medium. Cells at a final concentration of 10^3^ cells/well were seeded onto a micro slide glass (Matsunami, TF0808). After 24-hour incubation, the cells were fixed in a 4% paraformaldehyde solution for 15 min and washed three times with PBS for 5 min each. The cells were then treated with 0.2% Triton-X for 10 min and washed three times in PBS. Blocking was performed using 10% bovine serum albumin for 30 min before the primary antibody was applied and incubated at room temperature for 3 h. The primary antibodies used were against pan-cytokeratin AE1/AE3 (1:100 dilution; Novus Biologicals), vimentin (1:500 dilution; Santa Cruz Biotechnology), Wilms' tumor suppressor gene 1 (WT1) (ready-to-use; Dako), and cytokeratin 5 (CK-5) (1:400 dilution; GeneTex). Finally, a fluorescence-labeled secondary antibody (anti-mouse IgG antibody conjugated with Alexa Fluor 488, 1:500 dilution; abcam) was added and incubated at room temperature for an hour. Nuclei were counterstained with Hoechst (1:1000 dilution). The images were obtained by a fluorescence microscope (Olympus).

### 2.4. Xenograft models

To assess the tumorigenic capacities of cells in the effusion and establish an *in vivo* experimental model for future studies, two xenograft models of canine pericardial mesothelioma were generated by injecting a cell suspension (case 1, MC18003; passage at 0) at different sites. Two mice with severe combined immunodeficiency (SCID) were sacrificed (Animal ethical approval No. 29-92). SCID mice were injected with cell suspension (1 × 10^6^ cells in 50 μl PBS) subcutaneously (SC mouse) or intraperitoneally (IP mouse). The mice were monitored for changes in health conditions, such as gross appearance, body condition, breathing status, clinical behavior, and growth of any visible mass. When a visible mass developed in an SC mouse, both mice were euthanized with isoflurane, and a necropsy was performed. Subcutaneous and intraperitoneal masses found during necropsy were histopathologically analyzed. The tissues were fixed in 10% neutral buffered formalin, embedded in paraffin, and stained with hematoxylin and eosin (H&E) in a routine manner. The images were captured by a light microscope (Olympus).

### 2.5. Chemosensitivity assay

A chemosensitivity assay was performed to assess and compare the antitumor effects of different chemotherapeutic drugs on canine mesothelioma cells. Three primary cultures generated from pleural effusion in this study were used (MC18003, MC19002, MC19009; passage at 2–6). Two chemotherapeutic agents conventionally used for canine mesothelioma, carboplatin and doxorubicin, and two other chemotherapeutic agents used for human mesothelioma and available for veterinary patients, vinorelbine and gemcitabine, were tested as single agents. Carboplatin, doxorubicin, and vinorelbine were tested at 0.1, 1, 10, and 100 μM, whereas gemcitabine was tested at 1, 10, 100, and 1000 μM. Moreover, the effects of combination treatment with carboplatin and gemcitabine, a platinum-based agent and an antimetabolite, respectively, were assessed, considering that the first-line chemotherapy for human mesothelioma had been a combination therapy using a platinum-based antineoplastic and a novel antimetabolite. Gemcitabine was tested at different concentrations ranging from 0 to 1000 μM, whereas the concentration of carboplatin was fixed at either 10 or 100 μM. The chemotherapeutic drugs used in this study were those for clinical use. As such they were water soluble and a drug in powder was firstly dissolved in sterile saline followed by dilution to each concentration with culture medium. Control groups were cultured in culture medium only.

Approximately 5000 cells per well in 100 μl culture medium were seeded to a 96-well microplate for the chemosensitivity assay. Following 24-hour incubation and washing in PBS, a 100 μl culture medium containing a chemotherapeutic agent was dispensed. The cells were incubated with a chemotherapeutic agent for 72 h before a cell viability assay. For combination treatment, cells were initially treated with gemcitabine for 4 h, followed by treatment with carboplatin for 68 h. Cell viability was measured using the Cell Counting Kit-8 (CCK-8) (Dojindo) according to the manufacturer's instructions. Briefly, 10 μl CCK-8 solution was added to each well. After 2 h of incubation, the absorbance at 450 nm was measured using a microplate reader. Cell viability was calculated and compared to that of the non-treatment control. The assay was performed in duplicate for technical replicates and repeated three times on different occasions for biological replicates.

### 2.6. Statistical analysis

The average (mean) and standard error (SE) were calculated, and the differences between samples were examined. Kruskal–Wallis test was performed for statistical analysis, followed by the Steel–Dwass test for *post-hoc* analysis. Statistical significance was set at *p* ≤ 0.05.

## 3. Results

### 3.1. Clinical cases of canine pericardial mesothelioma

**Figure 1:**
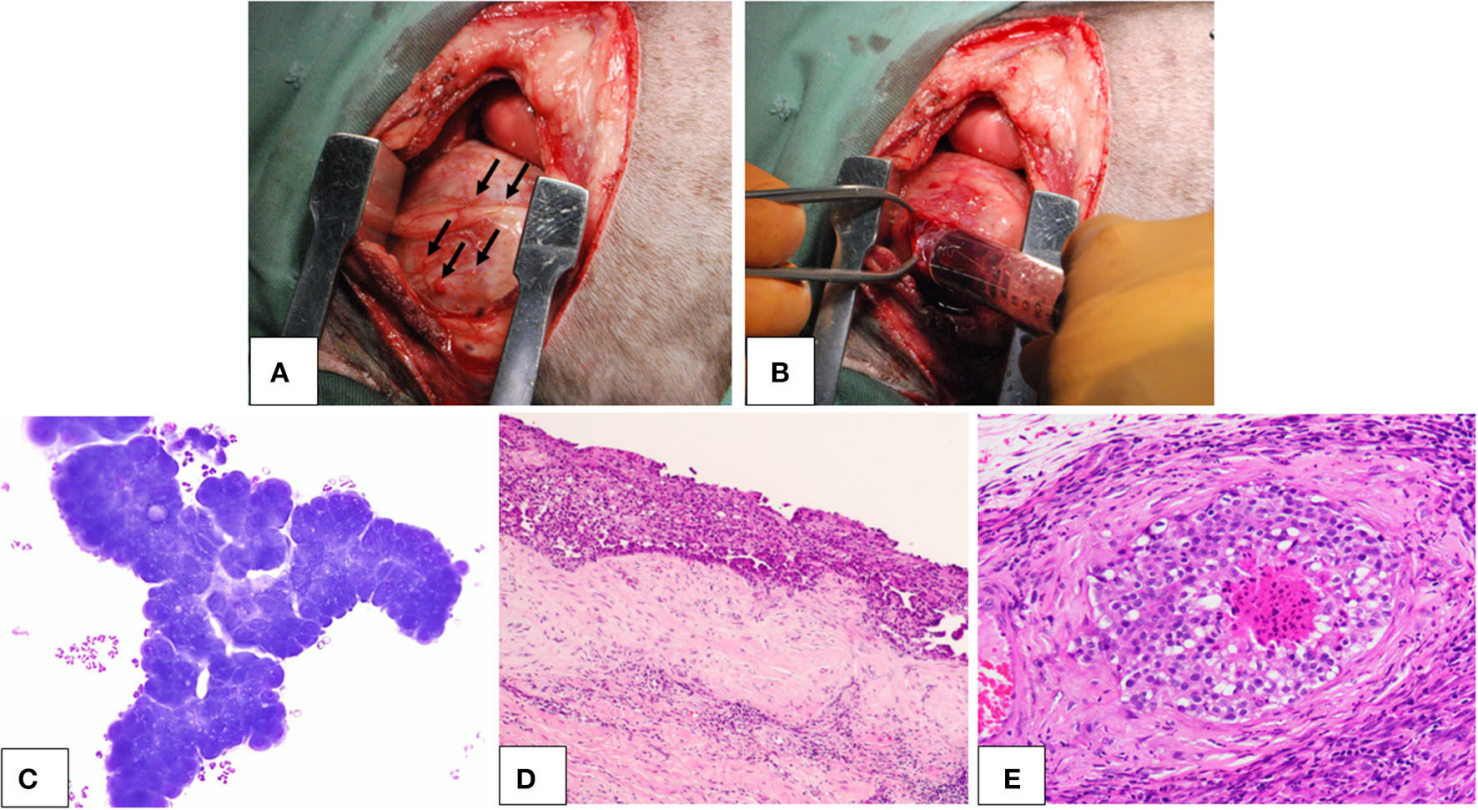
Canine pericardial mesothelioma. Gross lesions, cytology, and histopathology. **(A, B)** Gross findings of the canine pericardial mesothelioma during surgery. **(A)** Small nodular lesions (arrows) were seen throughout the pleural cavity, including the pericardium and pleura. **(B)** During pericardiectomy, pleural and pericardial effusions with hemorrhagic appearance were collected. **(C)** Cytology of pericardial effusion in a dog with pericardial mesothelioma. Neoplastic cells were observed in clusters that showed marked cellular atypia. **(D, E)** Histopathology of canine pericardial mesothelioma at **(D)** low (×4) and **(E)** high (×200) magnification. Neoplastic cells aligned with the mesothelium proliferated in thick layers. The tumor showed evidence of tissue and vascular invasion, indicating its malignant nature.

Three dogs were diagnosed with pericardial mesothelioma based on the histopathology of the resected pericardium. Signalment of the dogs are presented in Table 1. All three dogs exhibited clinical signs attributable to mesothelioma, such as lethargy, anorexia, weight loss, dyspnea, and heart failure, including cardiac tamponade and right-sided heart failure due to effusion. They had no discrete large masses on imaging diagnosis, including computed tomography and ultrasonography, and multiple small nodules on the surface of the pericardium and pleura were found during surgery (Figures 1A, B). Effusion samples for cell culture contained numerous neoplastic cells (Figure 1C). Histopathology of the resected pericardium revealed a marked proliferation of neoplastic mesothelial cells and invasion into the vessels and deeper tissues (Figures 1D, E).

**Table 1:** Signalment of the dogs involved in this study.

**No**.	**Breed**	**Sex**	**Age**
Case 1	Yorkshire Terrier	Castrated male	6 y 10 m
Case 2	Miniature Dachshund	Castrated male	10 y 1 m
Case 3	Miniature Bull Terrier	Spayed female	8 y 2 m

### 3.2. Cell culture and immunostaining

**Figure 2:**
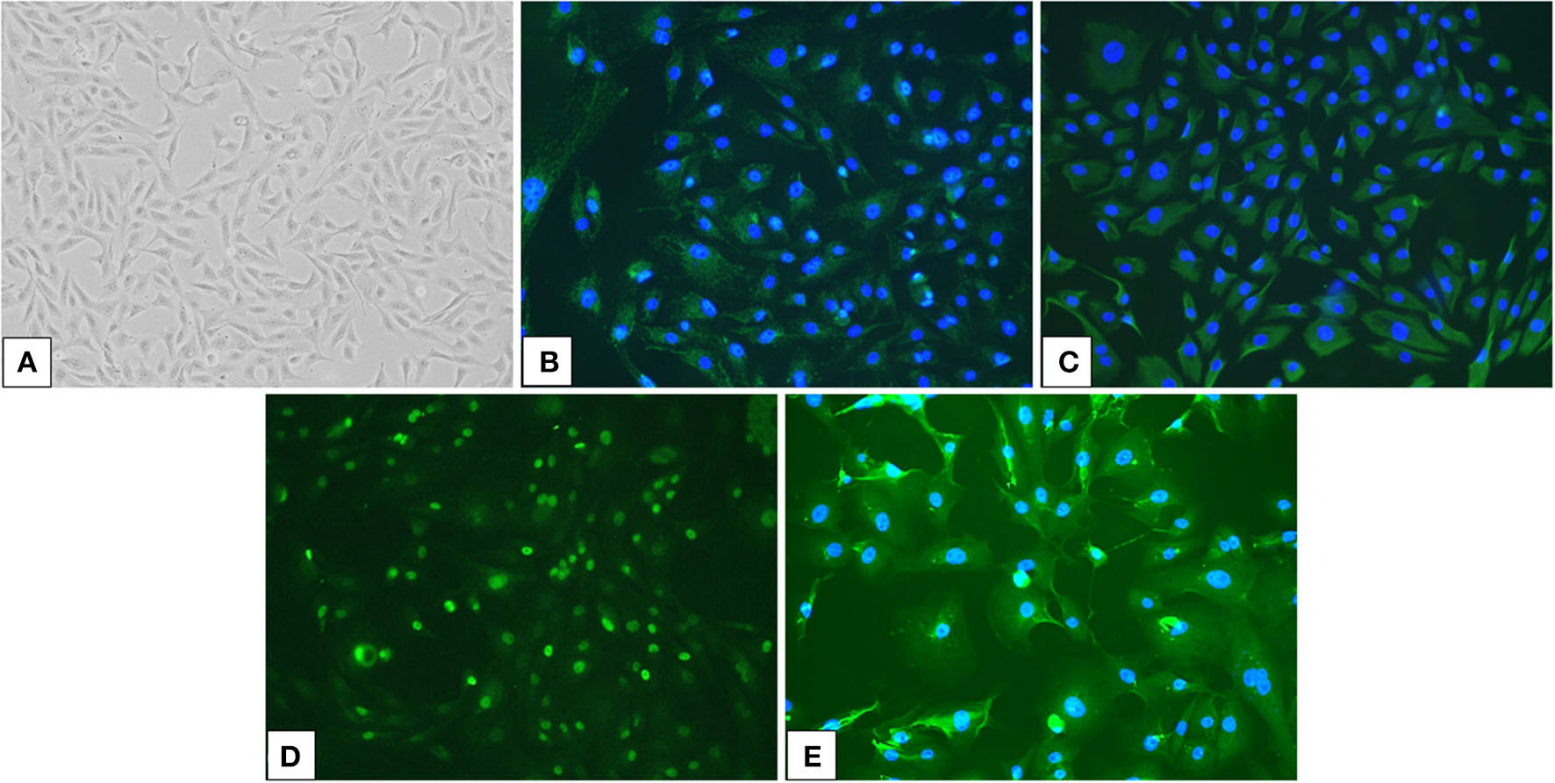
Culture cells isolated from an effusion sample from a dog with pericardial mesothelioma and their immunoreactivity for mesothelial markers. **(A)** Neoplastic cells in the pleural effusion from a dog with pericardial mesothelioma were isolated and cultured (×100). **(B, E)** Cultured neoplastic cells were double positive for epithelial markers **(B)** pan-cytokeratin (AE1/AE3) and mesenchymal marker **(C)** vimentin, as well as mesothelial markers **(D)** Wilms' tumor suppressor gene 1 (WT1) and **(E)** cytokeratin 5 (CK5) by immunofluorescence (×200).

Primary cell cultures were generated from the effusion samples. The cells in the culture formed monolayer sheets and grew in an epithelial pavement arrangement without contact inhibition (Figure 2A). The cells were polygonal to dendritic in shape and had round to oval nuclei and prominent medium-sized nucleoli, with occasional giant nuclei, binucleation, or multiple nucleoli. Immunostaining revealed that the cells were positive for AE1/AE3 and vimentin, which were epithelial and mesenchymal markers, respectively (Figures 2B, C). Moreover, the cells were positive for the mesothelial markers WT1 and CK5 (Figures 2D, E). The immunostaining pattern of WT1 was mostly nuclear, with weak cytoplasmic staining, whereas CK5 showed a cytoplasmic immunostaining pattern.

### 3.3. Xenograft models

**Figure 3:**
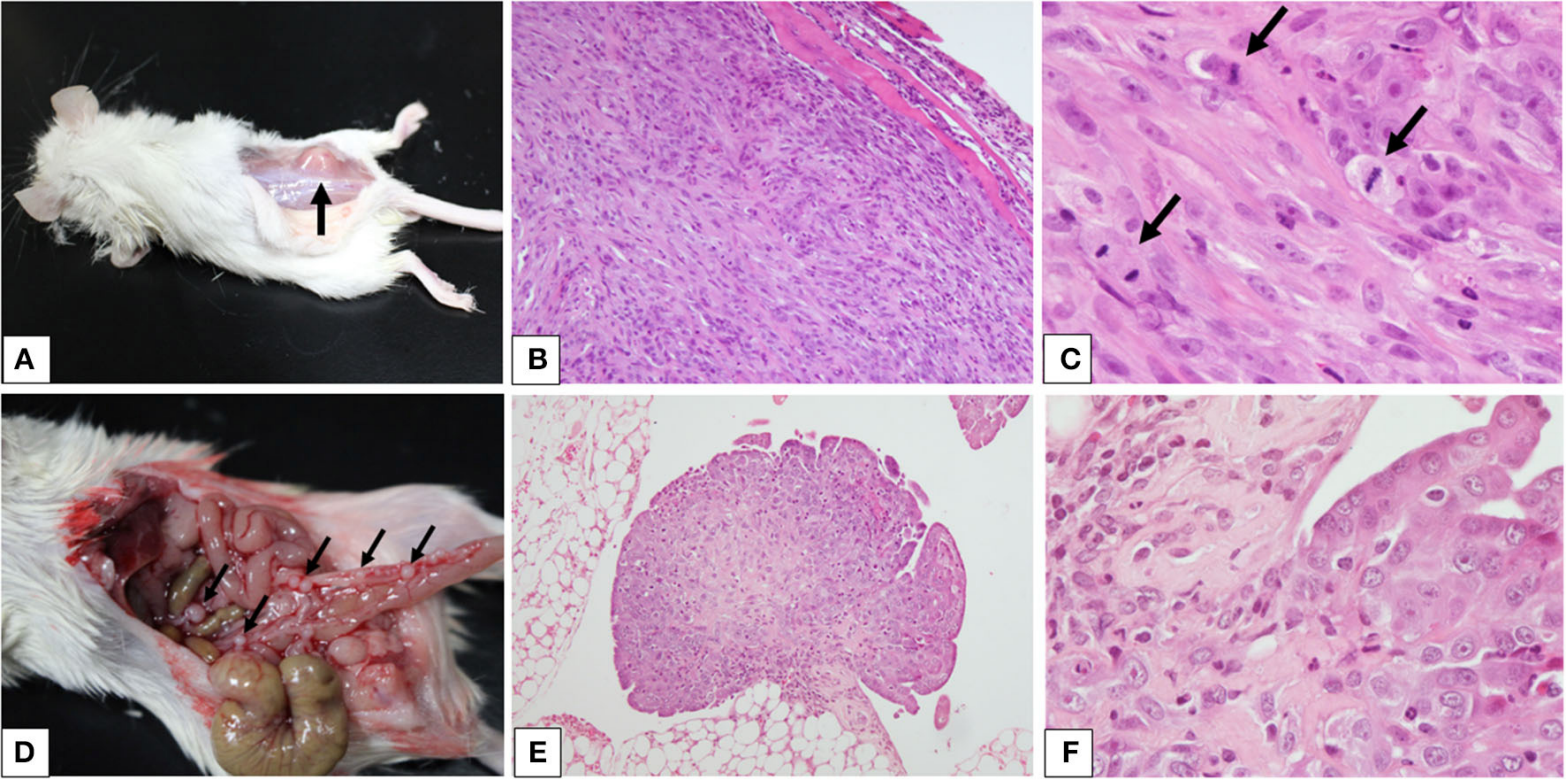
Xenograft models of canine pericardial mesothelioma using severe combined immunodeficient mice. **(A–F)** Xenograft models of canine pericardial mesothelioma were created by injecting neoplastic cells into the **(A)** subcutaneous tissue or **(D)** intraperitoneal cavity. The masses are indicated by the arrows. Histopathology of masses that developed in the subcutaneous tissue (B, ×100 and C, ×400) or intraperitoneal cavity [**(E)** ×100 and **(F)** ×400] revealed characteristic features of mesothelioma. High mitotic activity was observed [**(C)** arrows].

The SC mouse developed a visible mass 60 days after the injection of the cell suspension when a necropsy of both SC and IP mice was performed (Figure 3). Necropsy revealed a discrete, immobile mass in the subcutaneous to the muscular tissues where the cells were injected (Figure 3A). The mass was ~10 mm in diameter, firm, and white to tan in color on the cut surface. The IP mouse developed several small pedunculated nodules that varied in size (1–2 mm in diameter) on the serous membrane throughout the peritoneal cavity (Figure 3D). The nodules were discrete, dorm-like, exophytic, and white to tan in color.

Histopathology of the mass from the SC mouse revealed a well-demarcated, raised mass growing in the muscular layer (Figure 3B). The mass was composed of sheets of cohesive cuboidal to polygonal cells in the peripheral area, with bundles of spindle cells at the center. The polygonal cells had abundant eosinophilic cytoplasm with large, round to oval nuclei, finely stippled chromatin, and prominent nucleoli. The spindle cells had small to moderate amounts of eosinophilic cytoplasm with oval-to-elongated nuclei, coarse chromatin, and occasionally visible nucleoli (Figure 3C). Nodules from the IP mouse showed papillary and exophytic growth patterns (Figure 3E). They adhered to the serous membrane and invaded adipose tissue. The growth patterns and cellular characteristics were similar to those observed in the SC mouse (Figure 3F). These findings were consistent with mesothelioma.

### 3.4. Chemosensitivity assay

**Figure 4:**
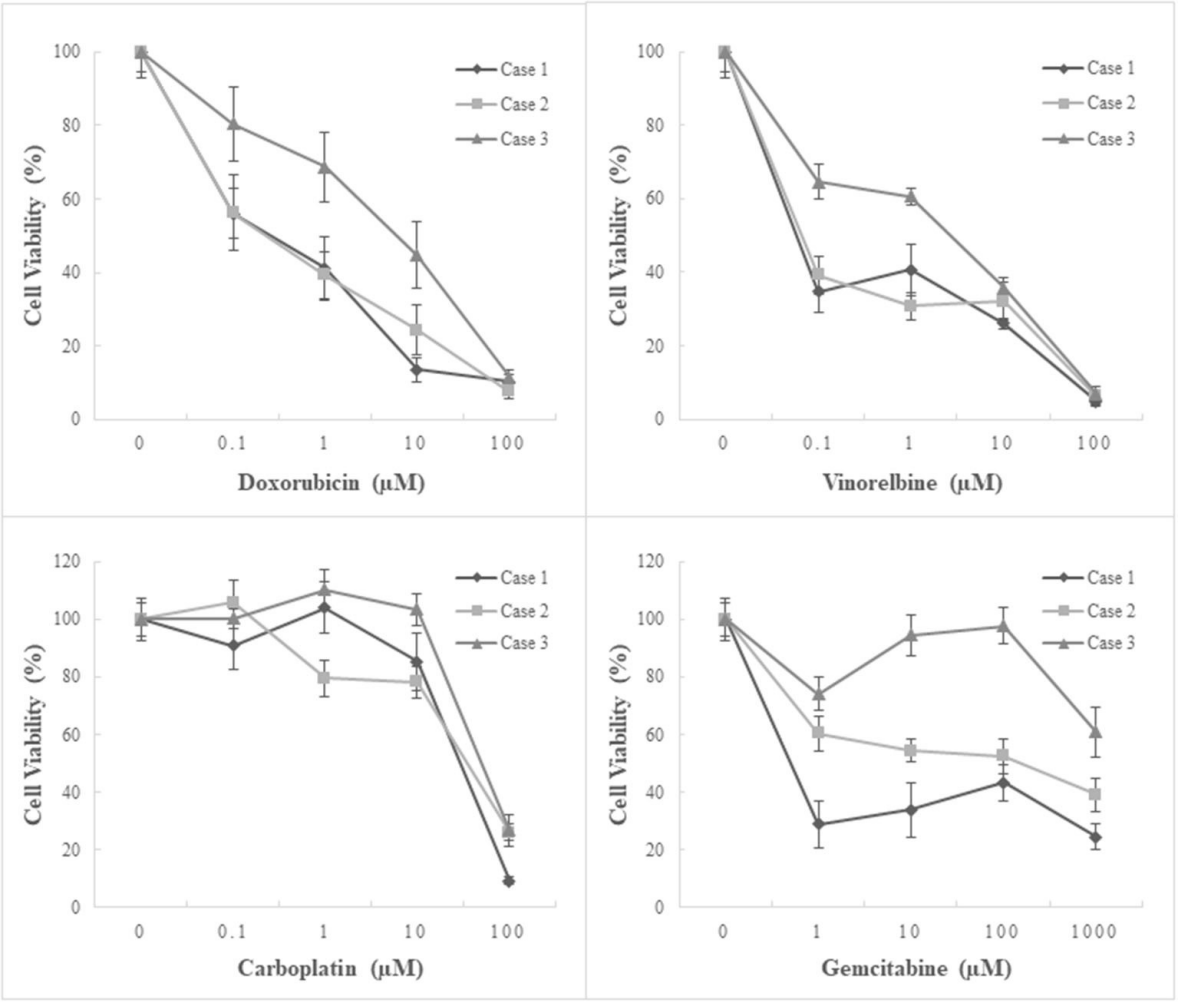
Chemosensitivity assay using three patient-derived primary cultures of canine pericardial mesothelioma. Single-agent treatment. Three patient-derived canine pericardial mesothelioma primary cultures were treated with four chemotherapeutic agents: doxorubicin, vinorelbine, carboplatin, and gemcitabine, as a single agent. Carboplatin demonstrated the highest inhibitory effects on cell growth among the four drugs, especially at clinically relevant concentrations.

**Figure 5:**
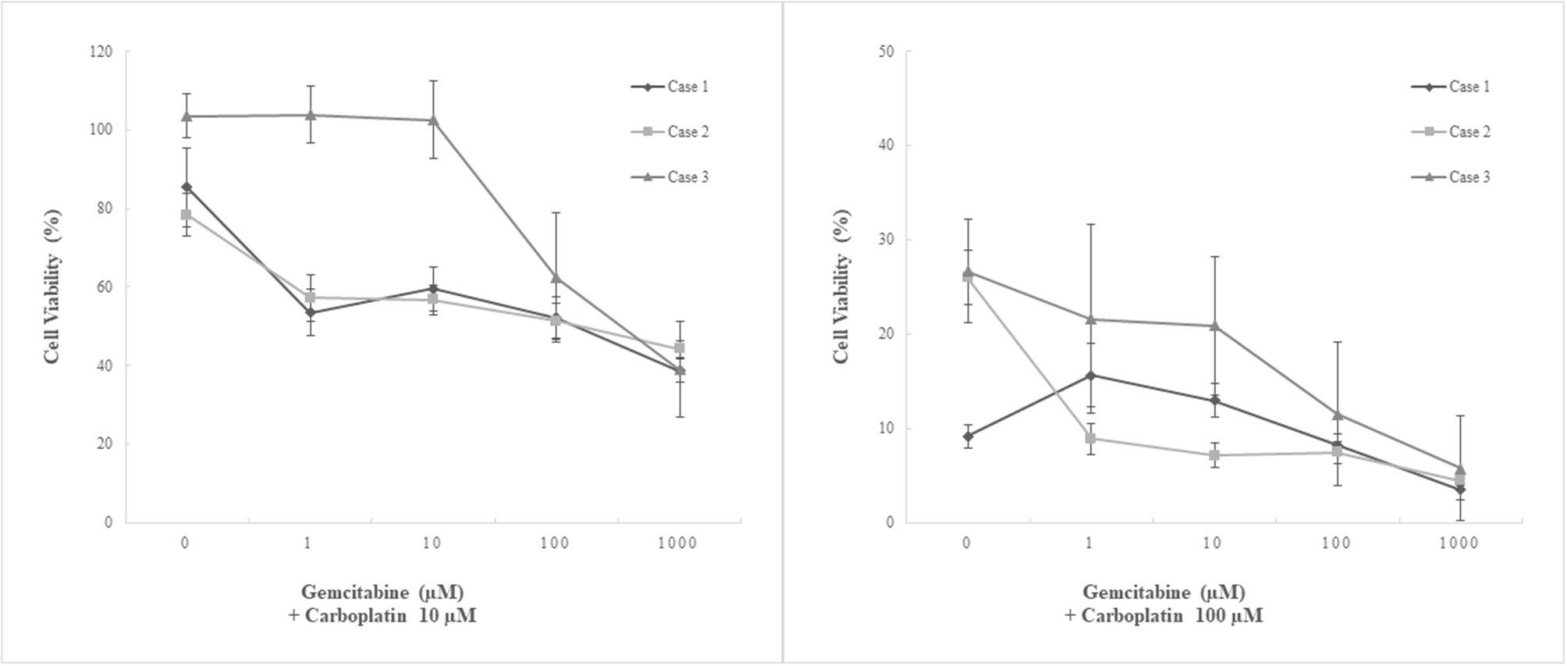
Chemosensitivity assay using three patient-derived primary cultures of canine pericardial mesothelioma. Combination treatment. Three patient-derived canine pericardial mesothelioma primary cultures were treated with a combination of two chemotherapeutic agents, carboplatin and gemcitabine. Carboplatin at 10 μM combined with any clinically relevant concentrations (<100 μM) of gemcitabine showed no augmented anti-tumor effects. On the other hand, carboplatin at 100 μM combined with ≥1 μM of gemcitabine significantly decreased cell viability in one cell line (case 2, MC19002) compared to single-agent treatment. Another cell line that showed relative resistance to carboplatin (case 3, MC19009) also tended to decrease cell viability when treated with 100 μM carboplatin and 100 μM or more gemcitabine; however, statistical significance was not detected.

The antitumor effects of the four chemotherapeutics on primary cultures of canine pericardial mesotheliomas were assessed using a cell viability assay as a single agent (Figure 4) or in combination (Figure 5). As for single-agent treatment, both doxorubicin and vinorelbine exhibited dose-dependent growth inhibitory effects. Compared to non-treatment control, doxorubicin inhibited cell growth to 64.3% (± 5.9) (mean ± SE), 49.7% (± 5.7), 27.5% (± 5.0), and 9.8% (± 1.2) on average at 0.1 μM, 1 μM, 10 μM, and 100 μM, respectively. Statistical significance was observed at ≥10 μM in all three cell lines (P ≤ 0.05). Similarly, vinorelbine inhibited cell growth to 46.2% (± 4.3), 44.0% (± 4.0), 31.3% (± 2.2), and 6.0% (± 1.0) on average at 0.1 μM, 1, 10 μM, and 100 μM, respectively, with a statistical significance detected at ≥1 μM in all cell lines (*P* ≤ 0.05). On the contrary to the previous two chemotherapeutics showing dose-dependent effects, carboplatin suppressed cell growth only at 100 μM, and it inhibited cell growth to 99.0% (± 4.3), 98.0% (± 5.4), 89.0% (± 5.0), 20.6% (± 2.9) on average at 0.1, 1, 10, and 100 μM, respectively. The statistical significance was detected at 100 μM in all three lines. The growth-inhibitory effects of gemcitabine were inconsistent between the cell lines. Two cell lines (case 1, MC18003; case 2, MC19002) were sensitive to gemcitabine at a dose of 1 μM, whereas the other cell line (case 3, MC19009) was resistant to the drug even at 1000 μM. Taking into account the peak plasma concentrations of each drug when administered clinically, doxorubicin at 0.5–1 μM, vinorelbine at 0.15–1 μM, carboplatin at 100–250 μM, and gemcitabine at 80–100 μM, which were equivalent ~30 mg/m^2^, 15–20 mg/m^2^, 300 mg/m^2^, and 675 mg/m^2^, respectively, were more relevant to clinical setting (16–22). Under these conditions, carboplatin was the most effective of the four drugs tested.

Combination treatment with carboplatin and gemcitabine was performed. Carboplatin at 10 μM, where single-agent treatment did not show inhibitory effects, suppressed cell growth significantly only when treated with gemcitabine at 1000 μM in combination. No augmented inhibitory effects were observed at clinically relevant concentrations of gemcitabine (≤ 100 μM). In contrast, carboplatin at 100 μM combined with ≥1 μM of gemcitabine significantly decreased cell viability in one cell line (case 2, MC19002) compared to single-agent treatment. Another cell line that showed relative resistance to carboplatin (case 3, MC19009) also tended to decrease cell viability at a dose of 100 μM or more of gemcitabine with 100 μM carboplatin; however, statistical significance was not detected.

## 4. Discussion

In this study, primary cultures of pericardial mesothelioma were successfully generated from pleural effusion of canine patients. Pleural effusions, rather than tumor tissues, were used because of their relatively easy accessibility and effectiveness in establishing cultures. Pleural effusion is observed when the delicate balance between the parietal and visceral pleura and pleural space is disrupted (23). Most mesotheliomas affect the parietal and visceral pleura and are often associated with pleural effusion. Cancer cells in the pleura produce inflammatory mediators, increase vascular permeability, and cause accumulation of fluids (i.e., effusion) in the pleural cavity (24). Mesothelioma cells can also be implanted and spread via cavitary spaces, invade deeper tissues and vessels, and obstruct lymphatic drainage, further facilitating the development of effusion (25, 26). In advanced stages of mesothelioma, effusion is usually classified as exudate or neoplastic effusion, containing numerous neoplastic cells from the tumor. Our samples also exhibited this characteristic, as mesothelioma cells at advanced stages tend to exfoliate readily into fluids (6, 23). Intriguingly, floating cancer cells retain the capability to form secondary foci, at least in part because of the support of nutrients and survival and mitogenic stimulators supplied by effusion (26). Moreover, dogs with pericardial mesothelioma often experience cardiac tamponade or dyspnea due to effusion of the pericardial and pleural spaces. Pericardiocentesis or thoracentesis is a palliative procedure to reduce the burden of effusion; collecting effusion samples for primary culture is an effective approach to collect cancer cells and a well-tolerated procedure with some benefits for a patient even at advanced stages where surgical intervention is no longer amenable. Furthermore, patients may undergo portal placement for the management of effusion and potential intracavitary chemotherapy, making effusion a feasible resource for culture (5, 6, 26, 27).

The cultured cells were characterized by immunostaining, which revealed immunoreactivity for both pan-cytokeratin and vimentin. These markers are routinely used for the initial screening of neoplastic tissues to classify epithelial and mesenchymal tumors. Because most cancers are typically labeled positively by either pan-cytokeratin or vimentin alone, positive staining with both markers often provides an initial clue for the potential diagnosis of mesothelioma. Thus, dual positivity is usually considered one of the characteristic features of mesothelioma, and both markers are traditionally used in combination for the presumptive identification of mesothelial cells, especially in veterinary medicine, where reliable antibodies are limited (1, 2, 6, 28–31). As a few other cancers can show dual positivity, antibodies specific to mesothelial cells, namely WT1 and CK5, were also used in our study. In humans, both are considered positive markers of mesothelioma (32, 33). WT1 is a transcription factor essential for the development of kidneys and gonads and has historically been known to be responsible for the tumorigenesis of Wilms' tumor, a rare renal tumor in humans (34). Alterations in this gene have been demonstrated in various tumors, and studies have shown that it may play an oncogenic role in these tumors (34–40). In human mesotheliomas, WT1 may promote cell proliferation, migration, and chemoresistance (41). A review that analyzed 88 published papers listed WT1 as one of the most specific diagnostic markers for mesothelioma with 96% specificity (42). Canine WT1 was molecularly cloned by Sakai et al. in 2017. The authors demonstrated the cross-reactivity of canine WT1 protein with a mouse monoclonal anti-human WT1 antibody (43). In this study, the same clone (6F-H2) of the WT1 antibody was used, which showed positive reactivity against primary cultured cells of canine pericardial mesothelioma. This result is consistent with a recent study that demonstrated the diagnostic utility of WT1 in canine mesothelioma (44). Positive labeling with the anti-WT1 antibody was strongly suggestive of the mesothelial origin of the cells. CK5 is another mesothelioma marker used in human medicine to differentiate mesothelioma from pulmonary adenocarcinoma (45–47). CK5 immunoreactivity has been detected in about 64–97% of mesotheliomas in effusions, cell block preparations, and histological sections (42, 45, 46). As the cultured cells were positive for WT1 and CK5, as well as dual positivity for pan-cytokeratin and vimentin, a mesothelial origin was confirmed.

Cells acquired from effusions also demonstrated tumorigenic properties when injected into a SCID mouse. The gross and histopathological findings of the masses that developed in SCID mice were similar to those seen in canine patients, including the clinical cases involved in this study (10, 25, 48–50). Mesotheliomas tend to develop as multiple small raised, nodular, papillary, miliary, or plaque-like lesions on the surface of the serous membrane rather than discrete large masses, as seen in typical malignant tumors. Considering this feature of mesothelioma, we generated two different types of xenograft models: SC and IP. SC models have been frequently created for *in vivo* studies of various cancers via relatively easy procedures such as subcutaneous injection of cell suspensions. A subcutaneous mass, if developed, could be palpated or assessed grossly so that when the mass has grown significantly, the endpoint of experiments can be determined to minimize the burden on an animal in terms of animal ethics (51). In this study, both mice were euthanized when a visible mass in the SC model reached a fair size, as it was difficult to assess intraperitoneal lesions and disease progression in the IP model; however, both mice behaved well clinically during the experiment. Additionally, a discrete visible mass is suitable for the assessment of anti-tumor effects during drug discovery or pre-clinical studies, which is one of the major purposes of *in vivo* experiments (52). Subcutaneous metastasis or seeding of mesothelioma is a rare complication of surgical intervention or thoracocentesis and has been sporadically reported in dogs with mesothelioma (3, 53). Thus, this SC model may be useful for understanding the mechanisms of subcutaneous metastasis or seeding of mesotheliomas and for investigating optimal treatment options. In contrast, the IP model recapitulates typical mesotheliomas, forming miliary nodular lesions on the serous membrane throughout the affected body cavity. As treatments for canine mesotheliomas often include intracavitary chemotherapy as the sole method or in combination with intravenous chemotherapy (11), this IP model could be suitable for understanding the biology and pathogenesis of the tumor, in addition to assessing the effectiveness of intracavitary and intravenous chemotherapy or new therapeutic modalities in a more appropriate tumor microenvironment. A major disadvantage of the IP model over the SC model is the difficulty in assessing tumor development, disease progression, and responsiveness to a drug or therapeutic modality of interest. Thus, researchers must have a special tool, such as positron emission tomography-computed tomography (PET-CT), as in the study by Collin et al., which provides limited availability to researchers in the veterinary field (54). Alternatively, the weight of the tumors and the volume of effusion may be assessed if the experimental endpoint was predetermined (55).

As canine mesotheliomas tend not to form discrete masses and are often diagnosed at advanced stages where complete surgical resection is unamenable, chemotherapy is the mainstay of treatment (11). Nevertheless, substantial preclinical studies on the effects of chemotherapeutic agents on canine mesotheliomas are lacking because of the unavailability of basic research tools such as canine mesothelioma cell lines. To address this problem, we first generated primary cultures utilizing clinical samples and used these cultures for *in vitro* studies to investigate the antitumor effects of chemotherapeutic agents on canine mesotheliomas. The four drugs were selected for testing based on previously published canine and human studies. Platinum-based drugs, such as cisplatin and carboplatin, and cytotoxic antibiotics, including doxorubicin, have been used to treat mesotheliomas in veterinary patients via either an intracavitary or intravenous route or both (11–15). The other two drugs investigated in this study, vinorelbine and gemcitabine, have often been used in human patients after they relapse or demonstrate chemoresistance against a first-line regimen consisting of a platinum-based agent, cisplatin or carboplatin, combined with a novel anti-metabolite (56–58). These two drugs have an advantage over other drugs in that they are available for veterinary patients with some degree of fundamental studies completed (20, 59–63). Our results support the historical use of carboplatin as the first-choice single-agent chemotherapy for canine mesothelioma, as it demonstrated the highest growth-inhibitory effects at clinically relevant doses among the four tested. Carboplatin, in particular, was of interest due to its relatively low toxicity compared to cisplatin. Because many patients with mesothelioma are middle-aged to old, they often have comorbidities that limit the use of cisplatin, which is highly toxic. However, cisplatin remains an attractive treatment option when no limitations exist for its use given their similar mechanisms of action as a platinum-based chemotherapeutic agent. Interestingly, the potential efficacy of cisplatin on canine mesotheliomas was suggested by a recent study (64). In the same study carboplatin also exhibited considerable degrees of anti-tumor effects, encouraging the continued clinical use of platinum-based chemotherapeutic agents. Doxorubicin was considered appropriate for use given its moderate activity, as shown in this study, in line with previously reported clinical outcomes (11, 13). Vinorelbine is unique in that it can be administered either intravenously or orally (20). Although its clinical use in veterinary patients is limited, vinorelbine may be a reasonable candidate for second- or third-line single-agent chemotherapy. However, further preclinical and clinical studies are required to validate these results.

Recently, therapeutic strategies for human mesothelioma have markedly evolved due to the introduction of several new anti-cancer drugs, including novel antimetabolite (57, 58, 65). Until 2004, single-agent chemotherapy with platinum-based agents was the first-line treatment for unresectable mesotheliomas. Since then, extensive studies on the novel antimetabolite have been conducted, and its high response rate when combined with platinum-based drugs has led to a change in the standard protocol to combination chemotherapy consisting of platinum-based agents and antimetabolite, which was approved by the U.S. Food and Drug Administration (57, 58, 65). We investigated the potential utility of combination chemotherapy using carboplatin (a platinum-based agent) and gemcitabine (an antimetabolite). Gemcitabine was chosen because of its relative accessibility to veterinary patients and its known synergism with platinum-based agents. The mechanisms underlying these synergistic effects are not fully understood; however, impaired deoxyribonucleic acid (DNA) repair and increased platinum-DNA adduct formation have been speculated (66–69). In this study, augmented growth-inhibitory effects were observed in cell lines that showed relative resistance to carboplatin after single-agent treatment. Interestingly, gemcitabine reverses resistance to cisplatin, another platinum-based agent, in several human cancers (70). Platinum-based agents work by forming DNA adducts with platinum to form intra- and inter-strand linkages that alter DNA structure, inhibit replication and transcription, and ultimately lead to cell death (71). DNA repair is a significant determinant of sensitivity to platinum-based agents, and resistance is often related to functional DNA repair systems (71). Gemcitabine may restore platinum sensitivity in cancer cells by impairing the DNA repair system. Gemcitabine incorporates its metabolites into the DNA, leading to DNA polymerase termination. The incorporation happens with an extra nucleotide following the metabolite, preventing excision by DNA exonucleases, a crucial DNA repair enzyme (66, 69). Gemcitabine also inhibits ribonucleotide reductase, an important enzyme in DNA replication and repair (66, 69). Moreover, the distorted DNA structures created by gemcitabine are favorable for binding platinum to DNA, which is a vital mechanism of action of carboplatin (66). This advantageous interaction between carboplatin and gemcitabine makes these two drugs promising candidates for combination therapy. Combination treatment with carboplatin and gemcitabine showed effectiveness against chemo-resistant cell lines in our study. This suggests that using this combination as a potential rescue protocol, or as a second-line treatment for refractory cancers previously treated with single-agent chemotherapy, could be a reasonable option. Further studies are warranted to evaluate the clinical utility of this protocol in canine patients.

In conclusion, this study demonstrated the feasibility of using primary cultures generated from effusions of dogs with spontaneously developed pericardial mesotheliomas. It provides researchers with fundamental tools for *in vitro* and *in vivo* studies. Furthermore, our results may contribute to the more rational use of anticancer drugs, such as platinum-based agents, in single-agent or combination chemotherapy against canine mesotheliomas.

## Data availability statement

The original contributions presented in the study are included in the article/supplementary material, further inquiries can be directed to the corresponding author.

## Ethics statement

The animal studies were approved by the Clinical Research Ethics Committee of the Tokyo University of Agriculture and Technology (no. 0016017). The studies were conducted in accordance with the local legislation and institutional requirements. Written informed consent was obtained from the owners for the participation of their animals in this study.

## Author contributions

RN: Conceptualization, Formal analysis, Investigation, Methodology, Resources, Visualization, Writing—original draft, Writing—review & editing. AK: Investigation, Resources, Writing—review & editing. ME: Investigation, Methodology, Writing—review & editing. TUs: Conceptualization, Data curation, Investigation, Project administration, Resources, Supervision, Writing—review & editing. TF: Conceptualization, Investigation, Supervision, Writing—review & editing. KS: Conceptualization, Formal analysis, Investigation, Methodology, Writing—review & editing. TUc: Conceptualization, Formal analysis, Funding acquisition, Investigation, Methodology, Project administration, Resources, Supervision, Validation, Writing—review & editing.
